# *mab-31 *and the TGF-β pathway act in the ray lineage to pattern *C. elegans *male sensory rays

**DOI:** 10.1186/1471-213X-10-82

**Published:** 2010-08-05

**Authors:** Yan-Fung Wong, Qing Sheng, Janet WL Chung, Jacky KF Chan, King L Chow

**Affiliations:** 1 Department of Biology, The Hong Kong University of Science and Technology, Clear Water Bay, Kowloon HONG KONG

## Abstract

**Background:**

*C. elegans *TGF-β-like Sma/Mab signaling pathway regulates both body size and sensory ray patterning. Most of the components in this pathway were initially identified by genetic screens based on the small body phenotype, and many of these mutants display sensory ray patterning defect. At the cellular level, little is known about how and where these components work although ray structural cell has been implicated as one of the targets. Based on the specific ray patterning abnormality, we aim to identify by RNAi approach additional components that function specifically in the ray lineage to elucidate the regulatory role of TGF-β signaling in ray differentiation.

**Result:**

We report here the characterization of a new member of the Sma/Mab pathway, *mab-31*, recovered from a genome-wide RNAi screen. *mab-31 *mutants showed ray cell cluster patterning defect and mis-specification of the ray identity. *mab-31 *encodes a nuclear protein expressed in descendants of ray precursor cells impacting on the ray cell's clustering properties, orientation of cell division plane, and fusion of structural cells. Genetic experiments also establish its relationship with other Sma/Mab pathway components and transcription factors acting upstream and downstream of the signaling event.

**Conclusion:**

*mab-31 *function is indispensable in Sma/Mab signal recipient cells during sensory rays specification. Both *mab-31 *and *sma-6 *are required in ray lineage at the late larval stages. They act upstream of *C. elegans Pax-6 *homolog and repress its function. These findings suggested *mab-31 *is a key factor that can integrate TFG-β signals in male sensory ray lineage to define organ identity.

## Background

Members of the transforming growth factor-beta (TGF-β) family are highly conserved multi-functional cell-cell signaling molecules found in many organisms [1,2]. The basic components of this pathway are secreted ligands, two receptor serine/threonine protein kinases (receptor types I and II), and the Smad (for *C. elegans *Small and *Drosophila *mad) proteins [3]. In worms, conventional TGF-β signaling pathways have been identified. They act downstream of two well characterized ligands DAF-7 and DBL-1 [4-6], which are referred to as the Dauer pathway and the Sma/Mab pathway, respectively.

The Sma/Mab pathway regulates body size and development of the sensory rays [7]. The secreted ligand encoded by *dbl-1 *triggers signaling events via *sma-6/daf-4 *receptors and Smad transducer molecules *sma-2*, *sma-3 *and *sma-4 *for downstream signaling [8,9]. Mutant animals with defective pathway are small in body size (Small) and mutant males have ray patterning defects in the tail (Male abnormal), i.e. fusion of ray pairs (4-5, 6-7, and 8-9) with different penetrance. While it is well established that the body size is a function of body hypodermis, less is known about the cellular context of the BMP signaling event in male sensory rays. Nonetheless, the wild-type sensory rays would need to express an array of genes to orchestrate developmental decisions that give rise to cells of distinct morphogenetic and functional identities, and dictate cellular positioning and assembly of individual organs.

Most components of the Sma/Mab pathway were identified by forward genetic approaches with a Sma phenotype [10]. More recent works using reverse genetics, DNA microarray analysis and yeast two-hybrid screens continue to identify modifiers, targets, and components of the pathways to uncover hitherto unknown biological roles of existing pathways and novel components [11,12]. Many of the newly identified *small *mutants, however, have no male tail defects at all [13-15]. These results suggest that distinct downstream signaling components may be required for male tail development functions to occur. We report here the characterization of a gene encoding a new TGF-β pathway component, *mab-31*, from a genome-wide RNAi screen. Animals with attenuated *mab-31 *activity displayed Mab phenotypes, specifically ray 4,5 fusion and 6,7 fusion. Through subsequent characterization of deletion mutants and genetic analysis, we demonstrate that *mab-31 *acts in Sma/Mab pathway to pattern specific male sensory rays. Epistatic analysis showed that the Sma/Mab regulatory cascade acts in the ray cell group and more specifically the ray structural cells, to attenuate the function of *Pax-6 *isoform, *mab-18*, during sensory ray development. Finally, we provide evidence that *mab-31 *encodes a nuclear protein working in the TGF-β signaling pathway and may represent a novel modulatory and context-dependent component acting in this sensory organ differentiation process.

## Result

### Male tail defect in *mab-31 *mutant resembles phenotypes in Sma/Mab mutants

**Figure 1:**
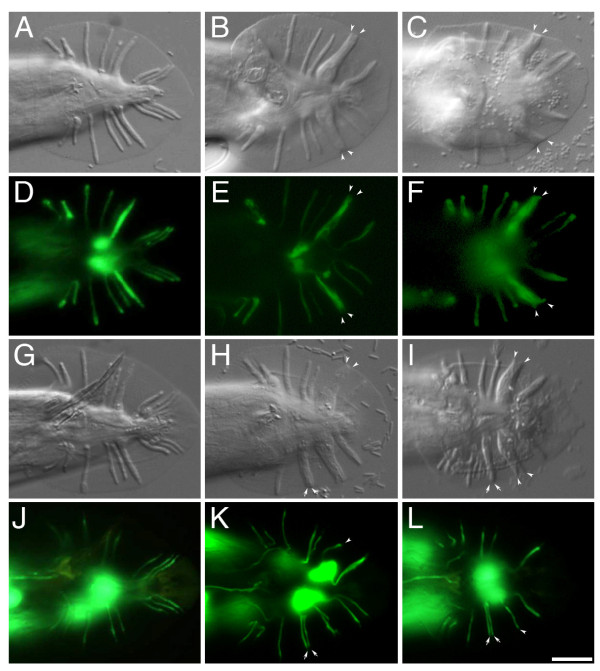
**Sensory ray patterns of mutants in Sma/Mab pathway**. Wild-type *C. elegans *male tail contains nine bilateral pairs of sensory rays (A and G). Their structural and neuronal (B type) processes were located by E1-GFP (D) and *ppkd-2::gfp2 *(J) markers, respectively. *sma-6 *(B and H) and *mab-31 *(C and I) mutants displayed rays 4-5 fusion (arrows) and rays 6-7 fusion (arrowheads) defects. In the defective rays, structural cell processes were fused (E and F), while the neuronal processes were separated (K and L). Ventral view. Scale bar = 20 μm.

*C. elegans *male tail is a fan-shaped structure (Figure 1A) required for copulation. It consists of nine bilateral pairs of peripheral organs known as sensory rays. Each ray consists primarily of a tube like hypodermal layer surrounding two sensory neurons and a glial-like structural cell [16]. Although sensory rays are similar in structure, each has a unique morphology and identity [17-19]. Each structural cell interacts with neurons from the same lineage and repels the neighboring structural cells and neurons [20]. Male tail defects in mutants of Sma/Mab pathway have been characterized as a transformation of ray identity [21], resulting in a high percentage of fusion in rays 4-5, rays 6-7, and rays 8-9 (Figure 1B). We isolated Mab mutants based on the specific abnormal sensory ray pattern, ray 4,5 and ray 6,7 fusion, through a genome-wide RNAi screen (Yip et al., in preparation). The RNAi bacterial clone I-7J10 from the Ahringer RNAi library could knock-down a nematode-specific gene, *Y54E10A.16*, on chromosome I. After the knockdown, male animals displayed a ray fusion phenotype and hermaphrodites showed a reduced fecundity. The gene harbored in this fragment has no previously characterized function and yet this knockdown gave a specific ray phenotype in male mutants, resembling that of the *dbl/sma *mutants.

The *Y54E10A.16 *gene is embedded in intron 9 of *cogc-1*, and the two genes are transcribed in opposite directions (Additional file 1). Null allele of *cogc-1 *did not show any male tail phenotype. The *Y54E10A.16(tm2718) *mutation kindly provided by NBRP, Japan, has a 435 bp deletion. This lesion is predicted to result in a truncated product lacking one-third of protein towards the C-terminus. *Y54E10A.16(tm2718) *mutants exhibit ray defects, with fusion of rays 4-5 (17%), rays 6-7 (60%) and rays 8-9 (31%) (Figure 1C) (Table 1, line 2). The ray patterning defects of *mab-31 are *reminiscent of those of *dbl-1, sma-6, daf-4, sma-2, -3, or -4 *(existing mutants in the Sma/Mab pathway) and displayed a penetrance matching the strongest ray phenotype of *dbl/sma *pathway mutations, e.g., null mutation of *sma-6*. We showed that the frequency of the ray fusion defects could not be enhanced further by feeding *tm2718 *males with I-7J10 RNAi bacteria. Such a result suggests that *tm2718 *behaves like a null mutation of *Y54E10A.16*. This gene is essential for ray patterning and display of the distinct male tail defect in mutant animals; the gene was designated as *mab-31*.

**Table 1:** Frequency of male tail sensory ray fusion in mutants

		Frequency of ray fusion (%)						
**Line**	**Genotypes**	**Ray 4**	**Ray 5**	**Ray 6**	**Ray 7**	**Ray 8**	**Ray 9**	**N^a^**

1	wild type (*mab-31 *RNAi)	3	3	10	10	10	10	240

2	*mab-31(tm2718)*	17	17	60	60	31	31	190

3	*sma-6 (wk7)*	21	21	38	38	16	16	250

4	*mab-31(tm2718);sma-6(wk7)*	20	20	61	61	25	25	270

5	*sma-6(e1482)*	0	0	0	0	3	3	180

6	*sma-6(e1482) (mab-31 RNAi)*	8	8	40	40	15	15	250

7	*mab-31(tm2718);sma-6(e1482)*	15	15	58	58	30	30	210

8	*mab-31(tm2718)*[p*mab-31*(2 kb)::*mab-31*cDNA::GFP]	0	0	0	0	5	5	132

9	*sma-6(wk7)*[p*mab-31*(2 kb)::*sma-6*cDNA]	8	8	16	16	13	13	150

10	*sma-6(wk7)*[p*mab-31*(2 kb)::*mab-31*cDNA]	20	20	36	36	15	15	210

11	*sma-4(e729)*	18	18	55	55	20	20	210

12	*sma-4(e729) *[p*mab-31*::*mab-31*cDNA]	17	17	53	53	21	21	200

Footnote: ^a ^the number of sides scoredFigure Legend.

Each wild-type ray consists of dendritic processes of two ultra-structurally distinct sensory neurons, RnA and RnB. The dendritic endings of these neurons are held at an opening to the environment by a support cell, i.e. the structural cell (Rnst) (Figure 1D). Structural examination of the abnormal ray in a ray-fusion mutant by electron microscopy showed the presence of a fused structural cell and separate neuronal processes [21]. We examined the cellular defect of the fused rays in *mab-31(tm2718) *males using two cell-specific reporters, E1-GFP [22] and *ppkd-2::gfp2 *[23], which mark Rnst and RnB cells, respectively. In both *sma-6(wk7) *(Figure 1E) and *mab-31(tm2718) *(Figure 1F), the structural processes were completely fused in 70% of defective rays at a frequency consistent with the observable defects revealed by DIC microscopy. The *ppkd-2::gfp2 *reporter was active in neuronal B processes from ray 1 to ray 9, but not ray 6 (Figure 1J). In both *sma-6 *and *mab-31 *mutants, the neuronal processes in the fused rays were well-separated (Figure 1K and Figure 1L). These results suggest that cellular defects in fused rays of both *sma-6 *and *mab-31 *mutants are similar. Abnormal fusion was observed only in extended distal processes of structural cells but not the neuronal processes. All existing mutants in Sma/Mab pathway with ray phenotype also displayed small body phenotype [24]. *mab-31(tm2718)*, however, is not Small and exhibits only a typical Sma/Mab ray fusion phenotype. The average body length of *mab-31(tm2718) *is 1.25 ± 0.07 mm (n = 100), in contrast to that of wild-type animals (1.23 ± 0.06 mm, n = 100) and *sma-6(wk7) *mutant (0.81 ± 0.03 mm, n = 100). *mab-31(tm2718) *mutation impacted other developmental processes also. For example, in mutant males, backward movement in response to an anterior touch is uncoordinated. They have crumpled copulatory spicules, which render the males unable to mate. The mutant hermaphrodites had a low brood size of (33.7 ± 15.4) as compared with wild-type (212.3 ± 31.5) [n = 20 for both]. These phenotypes are all specific, as they resemble those displayed by *mab-31(RNAi) *animals, and all abnormal phenotypes could be rescued by the functional transgene, p*mab-31(2 kb)::mab-31cDNA*.

### Specification of sensory rays requires both *mab-31 *and *sma-6 *at the L3/L4 larval stage

**Figure 2:**
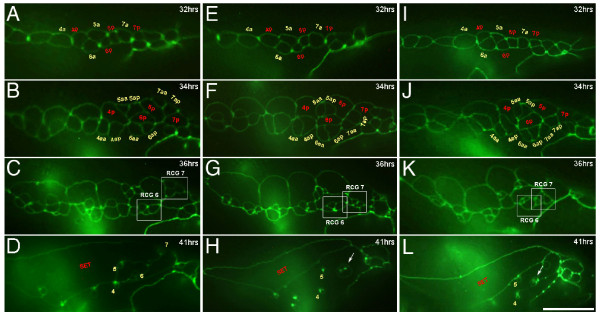
**Abnormal ray clustering patterns of mutants in Sma/Mab pathway**. The formation of daughter cells from ray precursor cells (Rn, n = 1-9) in different developmental stages was examined with apical junction markers *ajm-1*::*gfp*. Between mid-L3 and mid-L4 stage, Rn cells were divided by a stereotyped lineage pattern giving rise to ray cell groups (RCG) and were subsequently assembled (A-D) in wild-type animals. No abnormality is noted in the division of Rn.a and Rn.p cells in *sma-6 *(E) and *mab-31 *(I) mutants. In wild-type male, R7.aa and R7.ap are born at the dorsal side of R7.p (B). However, in *sma-6 *(F) and *mab-31 *(J) mutants, both R7.aa cells and R7.ap cells are skewed towards the ventral side of R7.p cell. The abnormal R7.aa and R7.ap reside next to the R6.aa and R6.ap cells. RCGs were well-separated in wild-type male tail (C) but not in both in *sma-6 *(G) and *mab-31 *(K) mutants. At a later stage, cellular components of ray 6 and ray 7 are clustered in close proximity and their structural cells are fused together (arrows in H and L). Lateral view, left side upwards. Hour (hrs) post-hatching at 20°C. Scale bar = 10 μm.

Ray identity determination is a dynamic process of ray precursor (Rn) cells differentiation in late larval stages of *C. elegans *male development. The underlying process of cellular specification of Rn cells is not well understood. Therefore, we examined and compared ray cell cluster profiles of wild-type, *sma-6(wk7)*, and *mab-31(tm2718) *animals. To achieve this, we traced the Rn cells lineage with an apical junction marker *ajm-1::GFP*. We were particularly interested in differentiation of R6 and R7, since rays 6-7 fusion was the most obvious phenotype in both *mab-31 *and other *sma *mutants. When R(6/7).a and R(6/7).p cells were born, there was no detectable difference in wild-type (Figure 2A), *sma-6 *(Figure 2E) and *mab-31 *mutants (Figure 2I). In the wild-type animal, R7.aa and R7.ap (descendents of R7.a) were produced along the A-P axis and stayed on the dorsal side of R7.p (Figure 2B). However, in *sma-6 *and *mab-31 *mutants, these two cells were present on the ventral side of R7.p after their birth (Figure 2F and 2J). In these mutants, the abnormal ray cell group derived from R7 sat next to that of R6 (Figure 2G and 2K) and subsequently, their structural cells were juxtaposed to each other and were fused together (Figure 2H and 2L). However, no cellular abnormality was observed in differentiation of R6a.a, R6a.p. or R6.p cells, in both mutants, as compared with those in wild-type animals. The defect in rays 6-7 fusion involved mis-localization of ray 7 precursors to ray 6 precursors during ray identity determination window at the L4 larval stage. Our observation suggested that both *sma-6 *and *mab-31 *mutants have a wild-type ray lineage with ray precursor cells born at the right time. The defects in these mutants were simply due to transformation of the ray cell identity, which dictates the cell cluster's positioning with respect to other ray cell groups. Indeed, similar defects were subsequently observed in other *sma *mutants (*sma-4 *and *dbl-1*, data not shown) also, inferring that the *mab-31 *gene probably acts in the same canonical *dbl/sma *pathway.

It was shown that the ray pattern in the adult male tail results from targeting of ray structural cell processes to specific sites, called papillae, in the L4 lateral epidermis [20]. Fused rays arise when multiple papillae are juxtaposed to each other in a cluster. Some of papillae were displaced in *mab-31(tm2718) *(65%) and *sma-6(wk7) *(45%) mutants (N = 50). For example, the ray 7 papillae in mutant animals were not found in their native position but often at an ectopic position just next to the ray 6 papillus. This displacement is also consistent with our observation that ray 6 and 7 cell groups were next to each other during ray development as described above (Figure 2G &2K). The same feature applies to papillae of ray 4 and 5, and to those of ray 8 and 9.

We further used a genetic approach to determine the functional relationship between *sma-6 *and *mab-31 *in the specification of sensory rays. Both *mab-31(tm2718) *and *sma-6(wk7) *carry recessive null mutations for ray patterning defects. We compared the frequency of rays 6-7 fusion phenotype in each single mutant as well as in double mutants. Worms doubly mutant for *mab-31 *and *sma-6 *do not exhibit an enhanced frequency of fusion for rays 6-7 (58%) compared to *mab-31 *single mutants (60%; Table 1). Thus, they are unlikely to be working in parallel for the ray 7 specification. The null allele of *sma-6(wk7) *showed both Sma and Mab phenotypes. However, in a weak loss-of-function allele *sma-6(e1482)*, the male tail defect could not be observed. It was suggested that the residual activity of *sma-6(e1482) *is sufficient for the male tail development, but its level is below the thresholds required for body size regulation [25]. Interestingly, a reduction of *sma-6 *activity in *e1482 *mutant background could enhance *mab-31(RNAi) *rays 6-7 fusion phenotype by four-fold, as compared with the same treatment in wild-type animals (Table 1, lines 1 & 6). This synergistic effect strongly argues that these two genes act in the same process and pathway. Furthermore, phenotypes of *mab-31(tm2718);sma-6(e1482) *double mutants resembled those observed in the *mab-31 *single mutant (Table 1, **lines 2 & 7**), suggesting that *mab-31 *is an indispensable component required for Sma/Mab pathway signaling during male tail development.

### MAB-31 may cooperate with SMAD inside nucleus

**Figure 3:**
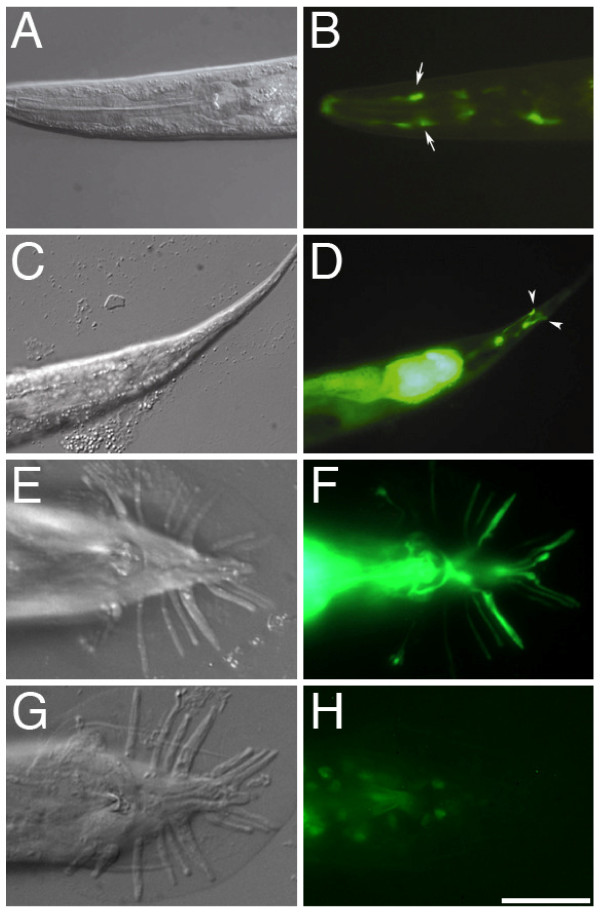
**Expression pattern of *mab-31***. The *gfp *expression from *mab-31::gfp *transcriptional reporter is found in amphid sockets (arrows) (B) and phasmid sockets (arrowheads) (D) in a hermaphrodite. In the male tail, *gfp *signal is detected in structural cell processes of all sensory rays (F). The functional MAB-31::GFP protein is localized in multiple ray cell nuclei (H). Lateral view, left side upwards for A-D. Ventral view for E-H. Scale bar = 20 μm.

In the Sma/Mab pathway, SMA-6 receptors transduce the extracellular signal to the effectors, SMA-2, SMA3, and SMA-4, which regulate the expression of downstream targets. Would *mab-31 *be one of the targets with expression regulated by the SMA-6 receptor and mediated by SMAD transcription factors in rays? To address this question, we first characterized the expression pattern of *mab-31 *gene. A 2 kb 5' upstream sequence of the *mab-31 *translational start was utilized for construction of a transcriptional *mab-31::gfp *reporter. The promoter sequence contained regulatory elements required for ray development, as it could be employed for expressing *mab-31 *cDNA to rescue both male tail (Table 1, **lines 2 & 8**) and other phenotypes in corresponding *mab-31(tm2718) *mutants. Indeed, the same 2 kb *mab-31 *5' upstream sequence could be used to drive expression of *sma-6 *cDNA and rescue *sma-6(wk7) *mutant ray phenotype (from ray 6,7 fusion drops from 38% to 16%, Table 1, **lines 3 & 9**). But the significant rescue was not observed in body length (0.82 ± 0.04 mm for *sma-6(wk7) *of the animals and 0.87 ± 0.06 mm for the same mutant with a transgene). This *mab-31 *2 Kb 5' franking sequence was active in gut cells of the pretzel-stage embryo and throughout the larval stages. In adult males and hermaphrodites, *gfp *reporter signal was observed in the pharynx, body hypodermis, and intestine. Interestingly, the type I receptor SMA-6 is expressed in all these tissues overlapping with the *mab-31 *pattern [25]. Yet, while *sma-6 *mutant animals have a small body phenotype, *mab-31 *mutants do not. Such a difference is probably due to the fact that *mab-31 *is not required nor expressed at a significant level at the early larval stage when *sma-6 *and *smad *functions are needed [26]. On the other hand, *mab-31 *expression is also observed in support cells of neuronal sensilla, like amphid socket cells (Figure 3B) and phasmid socket cells (Figure 3D), where no expression of *sma-6 *has been documented. In the wild-type male tail, the *mab-31 *transcriptional reporter expression was detected in structural cells highlighting the cell bodies and processes of all sensory rays (Figure 3F). Most importantly, the expression level was not changed in either *sma-6 *or *sma-4 *mutant backgrounds (data not shown). Indeed, high copies of *mab-31 *functional transgenes in ray structural cells cannot by-pass the requirements of either *sma-6 *or *sma-4 *in rays (Table 1, lines 3 & 10, 11 & 12). These observations suggest that *mab-31 *is not acting transcriptionally downstream of the Sma/Mab signaling cascade.

We explored the molecular function of MAB-31 in the Sma/Mab pathway by evaluating its cellular localization. MAB-31 is highly conserved across several *Caenorhabditis *species (Additional file 1), but it does not resemble any protein other than its worm counterparts. Based on the amino acid sequence, a stretch of polypeptide (RKRREK) was predicted as a nuclear localization signal (NLS) (PredictNLS online) implicating it to function inside the nucleus. To ascertain its *in vivo *subcellular localization, we generated transgenic animals carrying a MAB-31 translational fusion with GFP tagged at its C-terminus. This MAB-31::GFP protein was a functional equivalent to endogenous MAB-31, since it could fully rescue rays fusion phenotypes in *mab-31(tm2718) *(Rescue efficiency = 100%, N = 50) (Table 1, line 8). MAB-31:GFP was found to be localized in nuclei (Figure 3H), but not in cytoplasm in all developmental stages that we had examined. It was produced at the pretzel-stage and then in all expressing cells implicated by the transcriptional reporter. Localization of MAB-31::GFP protein was altered neither in *sma-4(e729) *nor in *sma-6(wk7) *mutants (N = 20 in both cases) throughout the ray differentiation period and in the adult stage. Thus, MAB-31 is likely acting constitutively in the nuclear compartment rather than being shuttled into the nucleus, like the Smad proteins.

### Sma/Mab signaling is modulated by epistatic interactions among *mab-18, mab-31*, and *unc-130 *in ray patterning

**Figure 4:**
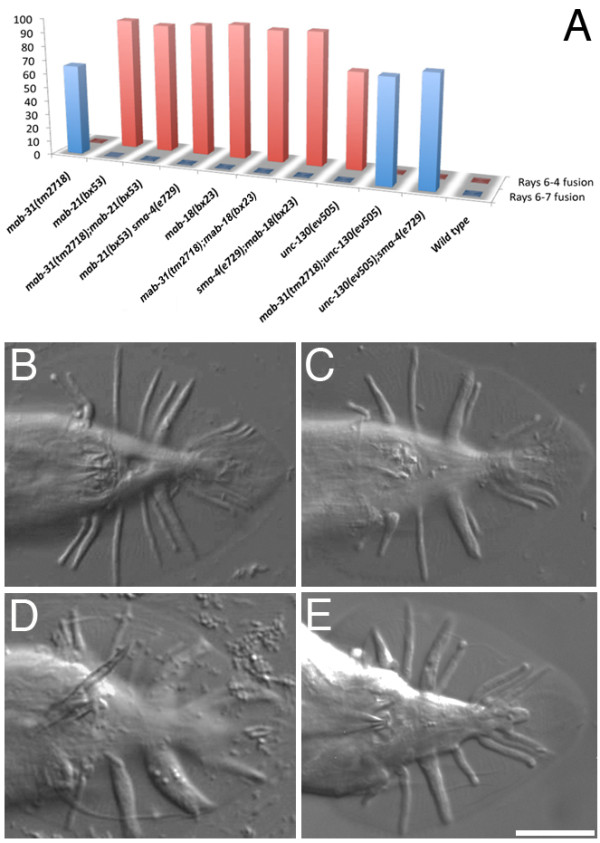
**Epistatic analysis of Sma/Mab pathway in ray 6 specification**. Frequency of ray fusion phenotypes (rays 6-7 fusion or rays 6-4 fusion) is shown in double mutants of *mab-31 *with those of Sma/Mab pathway (A) and with those showing rays 6-4 fusion phenotypes. In double mutants of *mab-31 *and *mab-18 *(E), only rays 6-4 fusion phenotype was observed, as compared with wild-type (B), *mab-18 *(C), and mab-31 (D) male tails. Ventral view for B-E. Scale bar = 20 μm.

Several components in Sma/Mab pathway were shown to negatively regulate the *mab-21 *gene in ray patterning [5]. In *mab-21 *mutants, the ray 6 structural cell apparently undergoes a specific transformation into a ray 4 morphogenetic identity and ray 6 is fused with ray 4. Double mutant of *mab-21 *with either *sma-6 *or *sma-4 *resulted in rays 6-4 fusion phenotype, which mimicked the Mab-21 phenotype. We could also establish a similar epistatic relationship between *mab-31 *and the components in *mab-21 *pathway. In the double mutant of *mab-31(tm2718); mab-21(bx53)*, only Mab-21 phenotype could be observed (Figure 4A). Thus the role of *mab-31 *is similar to Sma/Mab pathway, which negatively regulates *mab-21 *during the specification of ray 6.

Since the specification of ray 6 by Sma/Mab pathway could be used to dissect genetic function for *mab-31*, we extended our study to look for the epistatic relationship of *mab-31 *with other candidates. *Pax-6 *is a conserved transcriptional factor that plays a variety of roles during metazoan development, primarily in patterning elements of the nervous system [27]. The *C. elegans *pairbox-less *Pax6 *isoform, *mab-18*, acts cell-autonomously in ray 6 [28,29]. Loss-of-function *mab-18 *also results in transformation of ray 6 to ray 4 identity (Figure 4C). In double mutants of *mab-31 *and *mab-18*, only Mab-18 ray fusion phenotype was observed (Figure 4A and 4E). The same phenotype was also observed in double mutants of *mab-18 *with either *sma-4 *or *sma-2 *(data not shown, N>100). Thus, Sma/Mab pathway is proposed to have acted upstream of *mab-18*.

The fork-head transcription factor *unc-130 *functions in both ventral body muscle and male tail developments [30]. *unc-130 *acts as a negative regulator of *unc-129 *and works autonomously to repress *unc-129 *expression in ventral body muscles. In the male tail, *unc-130 *is required for keeping ray 6 identity independent of *unc-129*. *unc-130(ev505) *mutant displayed a rays 6-4 fusion phenotype of 82% of the animals. In the double mutant of *mab-31(tm2718);unc-130(ev505)*, only the rays 6-7 fusion phenotype was observed (Figure 4A). This finding suggests that *unc-130 *works further upstream of Sma/Mab pathway and negatively regulates *mab-31 *in the ray patterning. Based on all these observations, we propose a novel genetic cascade acting uniquely in the male tail sensory rays specification; *mab-18 *is the downstream target of *mab-31*. MAB-31 is localized in the nucleus, and we predict that it acts downstream of the SMA-6 receptor (and also DAF-4 receptor, data not shown), and this TGF-β signaling cascade is negatively regulated by *unc-130 *acting as the most upstream regulator in this process.

## Discussion

### Interpretation of the Sma/Mab signal by the ray lineage during male development

In this paper, we have reported the characterization of a nuclear factor encoded by *mab-31 *in the *C. elegans *for sensory ray patterning. *mab-31 *mutants present no small body phenotype, but exhibit male tail defects resembling the ray fusion phenotypes in existing Sma mutants of Sma/Mab pathway. We have shown that both MAB-31 nuclear factor and SMA-6 receptor are required to function in the ray lineage and the structural cells during the ray patterning process. They regulate the ray patterning event by altering the position of the R7 derived ray cell cluster and how it associates with the neighboring cluster at the late L4 males. Finally, we establish the genetic relationship of conserved transcriptional regulators in ray patterning, such as *unc-130 *and *mab-18*, with Sma/Mab pathway components through epistatic analysis.

In Sma/Mab pathway, the signaling ligand *dbl-1 *is expressed primarily in motor neurons along the ventral nerve cord in both hermaphrodites and males [5,6]. We have previously showed that ectopic expression of *Xenopus *BMP4 in these neurons could functionally rescue body size [15] and corrected the ray fusion defect (Wong and Chow, unpublished data) in *dbl-1 *mutant. Expression of the *dbl-1 *ligand in neuronal processes along the body is sufficient for its function in the ray patterning, probably through diffusion of this extracellular ligand. Hence, the specificity of Sma/Mab signaling for ray patterning is likely to be controlled by receptors residing in cellular components of the sensory rays but not by the source of the signal acting at a distance. However, expression patterns revealed by transcriptional reporters for TGF-β receptors and Smads did not reveal their expression in any specific cells within the sensory rays [8,25]. For example, hypodermal expression of *sma-6*, which is governed by a GATA sequence in the *sma-6 *promoter, was shown to be necessary for maintenance of body length [8]. This expression, however, was not sufficient to rescue the male tail mutant defect (Ho and Chow, unpublished data). In this report, we have shown for the first time that the *mab-31 *promoter with activity in the ray structural cell (Figure 3F) could functionally express *sma-6 *transgene and successfully rescue the ray fusion defect of *sma-6 *mutant (Table 1, **line 9**) even when the structural cell-specific *sma-6 *regulatory element remains undefined. This finding infers that both *mab-31 *and *sma-6 *likely have overlapping expressions in the same cells in the ray sublineage including the structural cells as part of the executing machinery of the *dbl-1 *signaling event. It is likely that their expression is required even earlier in the structural cell precursors R7.ap or R7.a. Without a good structural cell-specific promoter active at the early stage of this cell type or in its precursors, direct experimental verification of this notion would not be possible. Neither would we be able to rule out the possibility that simultaneous expression of *sma-6 *nor *mab-31 *in both hypodermis and structural cells is necessary for establishing correct ray identity. In addition, with the cellular defects revealed in *mab-31*, *sma-6 *and other *sma *mutants which converge on having an abnormal division plane of the R7.a, the action of the *dbl-1 *pathway receptors and relay molecules may indeed act even as early as the R7.a cell is born.

Examination of morphology of ray structural processes in fused rays of *mab-31, sma-6, sma-4, and dbl-1 *mutants also showed that fusion of structural cells but not fusion of neuronal processes within rays is the primary defect. This observation is similar to the results of the histological examination of the fused ray in *mab-21 *mutant, which implicates that fused structural cells are the primary cause of ray fusion after the ray identity was altered [21]. Collectively, these evidences support the notion that single ray formation acts through the ray lineage and manifested through assumption of a unique identity which can be manifested by the structural cells. The outcome is abnormal ray fusion, when structural cells of two adjacent rays lose specificity-determinants required to keep the rays apart as independent entities [16].

### Biological role of Sma/Mab signaling in sensory ray patterning

Multiple evidences have shown that Sma/Mab signaling in the structural cells is required in patterning of sensory rays. The attachment of the structural cells to specific positions of the epidermis and cuticle at late L4 stage dictates where the papillae will be formed. In turn, the ultimate position of a ray is determined [20]. When the attachments are altered in different mutants, such as in *sma *mutants or many *mab *mutants, ray patterning is changed [21,31]. However, the underlying process governed by Sma/Mab signaling on how structural cells are localized to distinct sites in the epidermis has not been characterized. Ray cell groups tend to reside at the junctions of two Rn.p cells. These hypodermal cells are generated at the first ray precursor cell division before the cell cluster patterning is obvious (Figure 2C). Ray 5 and 7 cell groups are born above their respective Rn.p cells while the others stay beneath the hypodermal daughter at birth. We have analyzed the arrangement and geometry of developing ray cell groups of the late L3 and L4 stages to examine how these sites are altered in mutants of Sma/Mab pathway. Our results demonstrate that positions of R6 derived cells are essentially normal in these mutants. Sma/Mab signaling is not involved in pre-patterning of ray 6 precursor cells. Similarly, formation of parental cells R7.a and R7.p in either *mab-31 *or *sma-6 *mutants did not differ from that in wild-type animals. However, in mutants, R7.a derivatives were abnormally positioned beneath the R7.p cells (Figure 2G and 2K), which may probably have been caused by the initial skewing of cell divisions leading to the positioning of R7.aa and R7.ap ventral to R7.p (Figure 2F and 2J). This feature indicates that in wild-type animals, Sma/Mab signaling event is required in the R7.a for proper formation of its daughter cells, R7.aa and R7.ap, possibly by skewing their division plane towards the dorsal side of R7.a. Such organization is maintained in wild-type individuals to separate the daughters of R6.a and R7.a over a distance by R5.p and R6.p. We therefore hypothesize that a novel mechanism of Sma/Mab signaling acts in sensory rays patterning. Receptors SMA-6 and DAF-4 are located at R7.a and are activated upon binding of the DBL-1 ligand, which is secreted from the ventral nerve cord. The signal is then transmitted through intracellular SMAD molecules and nuclear factor MAB-31, to its targets which are potentially required for establishment and maintenance of cell polarity of R7.a and thus identity of its descendants. Disruption of this signaling event may change the cellular fate of R7.a and its subsequent plain of division leading to an abnormal position of the daughter cells with respect to the neighboring cells. As a result, fusion of ray structural cells, a descendent of R7.a, is possible because of the loss of identity and physical proximity between the fusing partners. It ends up with a ray 6-7 fusion defect. Interestingly, in the absence of *mab-18*, this Sma/Mab signal is dispensable and the identity of ray 7 could be restored. Would the maintenance of ray 7 identity involve deregulation of *mab-18 *activity through the Sma/Mab signaling? That's certainly a possibility since MAB-18 subcellular localization in the ray 7 cluster is developmentally regulated [29]. Nevertheless, the precise molecular regulation involving MAB-31 and SMAD molecules demands a more detailed characterization. It will be an important prerequisite before the operation of a complete genetic network during sensory ray patterning can be addressed.

## Conclusion

*mab-31 *is identified to encode a nuclear protein that acts in the Sma/Mab pathway during *C. elegans *male sensory ray cell development. We have shown by genetics that *mab-31 *acts in Sma/Mab signal receiving cells in the ray lineage. Furthermore, we have demonstrated by epistatic analysis that the Sma/Mab pathway acts upstream of *mab-21 *and *mab-18 *within the ray lineage including the structural cells, and the entire pathway is negatively regulated by *unc-130 *upstream of the *dbl-1 *signals. These data collectively suggest potential cross-talk between ray cells with patterning signal acting via the diffusible DBL-1 and the involvement of important structural cell-specific molecules to mediate cell fate specification and subsequent differentiation.

## Methods

### Strains

Strains were maintained as described by Brenner [10]. To facilitate male tail analysis, each strain, including the *mab-31(tm2718) *mutant, was crossed with *him-5(e1490)V *to generate a double mutant (see below). Strain names and genotypes of animals used were:

**FX27180: ***mab-31(tm2718)I*,

**NF2990: ***cogc-1(k179)I*,

**CB40880: ***him-5(e1490)V*,

**KC8250: ***mab-31(tm2718)I; him5(e1490) V*,

**KC1260: ***sma-6(wk7)II; him-5(e1490)V*,

**KC5880: ***sma-6(e1482)II; him-5(e1490)V*,

**KC5470: ***unc-130(ev505)II; him-5(e1490)V*,

**KC4470: ***rrf-3(pk1426)II; him-5(e1490)V*,

**EM1280: ***mab-21(bx53)III; him-5(e1490)V*,

**KC5360: ***sma-4(e729)III; him-5(e1490)V*,

**EM660: ***him-5(e1490) V; mab-18 (bx23) X*,

**KC8720: ***mab-31(tm2718)I; dpy-17(e164) mab-21(bx53) III; him-5(e1490)V*,

**KC9250: ***mab-31(tm2718)I; sma-6(wk7)II; him-5(e1490)V*,

**KC10700: ***mab-31(tm2718)I; him-5(e1490)V; mab-18(bx23)X*,

**KC10710: ***mab-31(tm2718)I; unc-130(ev505)II; him-5(e1490)V*,

**KC8410: ***wxIs29(pRF4+pKS+T24C2sphIGFPNLS-)I; sma-6(wk7)II; him-5(e1490)V*,

**KC8430: ***sma-6(wk7)II; su93[jam1::GFP; pRF4; unc-29] JcIs1IV; him-5(e1490)V*,

**KC8420: ***sma-6(wk7)II; him-5(e1490) wxIs52[pB0024.14b::gfp+ppkd-2::gfp2]V*,

**KC8470: ***mab-31(tm2718) wxIs29(pRF4+pKS+T24C2sphIGFPNLS-)I;him-5(e1490)V*,

**KC8480: ***mab-31(tm2718)I;him-5(e1490)V;wxIs52[pB0024.14b::gfp+ppkd-2::gfp2]V*,

**KC8490: ***mab-31(tm2718)I; jcIs1[ajm1::gfp; pRF4; unc-29]IV; him-5(e1490)V*,

**KC8090: ***him-5(e1490)V; wxEx64[pRF4+pmab-31::gfp(2.0 k)]*,

**KC9640: ***him-5(e1490)V; wxEx123[pRF4+pmab-31(2 kb)::cDNA::GFP]*, and

**KC9650: ***mab-31(tm2718)I; su93[jam-1::GFP; pRF4; unc-29]JcIs1IV;him-5(e1490)V; wxEx122[pcrm-1b::gfp+pmab-31(2 kb)::cDNA::GFP]*

### RNAi

RNAi clone I-7J10 was from the Ahringer RNAi library (MRC Geneservice) [32]. The identity of this clone was determined by in house sequencing. RNAi bacteria cultivation and double-stranded RNA induction were performed as described by Kamath et al. [33]. KC447 worms were synchronized by hypochlorite treatment, and the resulting L1 animals were placed on bacteria spotted plates and incubated at 20°C. Male tail phenotypes were assessed in adult worms after 3 days. In all RNAi assays, *E. coli *HT115(DE3) carrying the empty RNAi vector L4440 was fed to the same strain as a negative control.

### Male tail phenotype characterization and body-length measurement

The male tails were examined by Nomarski microscopy (*N *> 100). Of the transgenic strains, only transformed worms carrying the markers were imaged and scored. For the body length measurement, L4 hermaphrodites grown at 20°C were transferred to fresh NGM plates. Five days later, 100 F1 adult hermaphrodites were randomly photographed under 5× or 10× objectives with SPOT RT camera (Diagnostic Instruments). Their body length was measured through a scale adjusted with SPOT RT v3.4.5 software and was presented as mean length with standard error.

### Lineage analysis

Worms were mounted on 2% agarose pads in 2 μl of M9 mixed with a smear of OP50 bacteria. A 12 × 12 mm cover slip was gently lowered onto the worm and divisions of the ray precursor cells marked by the expression of *ajm-1::GFP *reporter (Strain SU93) were observed and recorded over a period of 4-5 hours.

### Transgene construction and rescue experiment

A *mab-31 *translational GFP fusion construct was made using pPD vectors kindly supplied by the Fire Lab (Stanford University) and verified by sequencing. GFP from pPD95.75 was excised using *KpnI *and inserted into the *KpnI *site at the 3' end of *mab-31 *cDNA amplified from wild-type animals, with stop codon mutated. This GFP-tagged 3' *mab-31 *cDNA was inserted into a vector carrying 2 kb of *mab-31 *upstream sequence, which is amplified with primers KC 1058 (5' GCTCGAATAACCTGG 3') and KC1059 (5' AGGTCCGCCCACTTGTT CC 3'), to generate a full-length *mab-31 *cDNA GFP-tagged rescuing construct, MAB-31A::GFP. The transcriptional reporter *mab-31::gfp *was generated by utilizing the same 5' upstream sequence and cloned into pPD95.75 vector. Germ line transformation with different constructs was carried out as described [34]. All transgene plasmids were co-transformed with either the dominant *rol-6 *gene or *crm-1b::gfp *as the selection marker. In general, at least three transgenic lines were generated for each plasmid construct and rescue or expression data were collected and pooled for comparison.

### Outcross of *mab-31(tm2718) *and generation of double mutants

*mab-31(tm2718) *was outcrossed with *him-5(e1490) *three times. Homozygosity of *mab-31 *mutation was confirmed by PCR using primer sets (KC1599 5' ATGAACTCGCTCGAGTACC 3' and KC1601 5' GGTACCTTCTGATAAAGCAA ACGG ATTA 3'). Double mutants containing mutations in *sma-4, mab-18, mab-21, unc-130 *and *mab-31 *were constructed by crossing *mab-31(tm2718) *heterozygote males with homozygous hermaphrodites of each mutant. For double mutants with either *sma-4 *or *unc-130*, Sma or Unc mutants were carefully counted and placed onto separate plates. Their *mab-31 *genotypes were confirmed by PCR. Sma or Unc hermaphrodites that bore a *mab-31(tm2718) *homozygous gene were selected as double mutants. For double mutants with either *mab-18 *or *mab-21*, F2 hermaphrodites were placed onto separate plates and their *mab-31 *genotypes were confirmed by PCR after F3 progeny were laid. The plates bearing males with Mab-21 or Mab-18 phenotype were confirmed as double homozygous mutants.

## Authors' contributions

YFW, QS, and JWC contributed equally in conducting most of the experiments in this study. JKC examined the cellular defect of Sma/Mab mutants and carried out lineage analysis. YFW and KLC supervised the project and prepared the manuscript. All authors have read and approved the final manuscript.

## Supplementary Material

Additional file 1 **Schematic representation of *mab-31 *gene structure and protein sequences**. *mab-31 *gene products are embedded in ~10.5 kb intron 9 of *cogc-1*. Mutation *tm2718 *deleted coding sequence of *mab-31a*, but it does not affect the transcript level of *mab-31b*, a transcript irrelevant to the ray fusion phenotype. B) Sequence alignment of nematodes MAB-31 proteins (*C. elegans*: WP_CE24445, *C. briggsae*: BP_CBP03618, *C. remanei*: RP_RP22498, and *C. brenneri*: CN_CN37332) was shown by ClustalX. The conserved nuclear localization signal (NLS) (PredictNLS online) and deleted region of MAB-31 protein in *tm2718 *mutant were predicted and labeled. Click here for file
