# Supplementary figures and images for: mab-31 and the TGF-β pathway act in the ray lineage to pattern C. elegans male sensory rays
Source: BMC Dev Biol. 2010 Aug 5;10:82. doi: 10.1186/1471-213X-10-82 (PMC2921377; doi:10.1186/1471-213X-10-82)

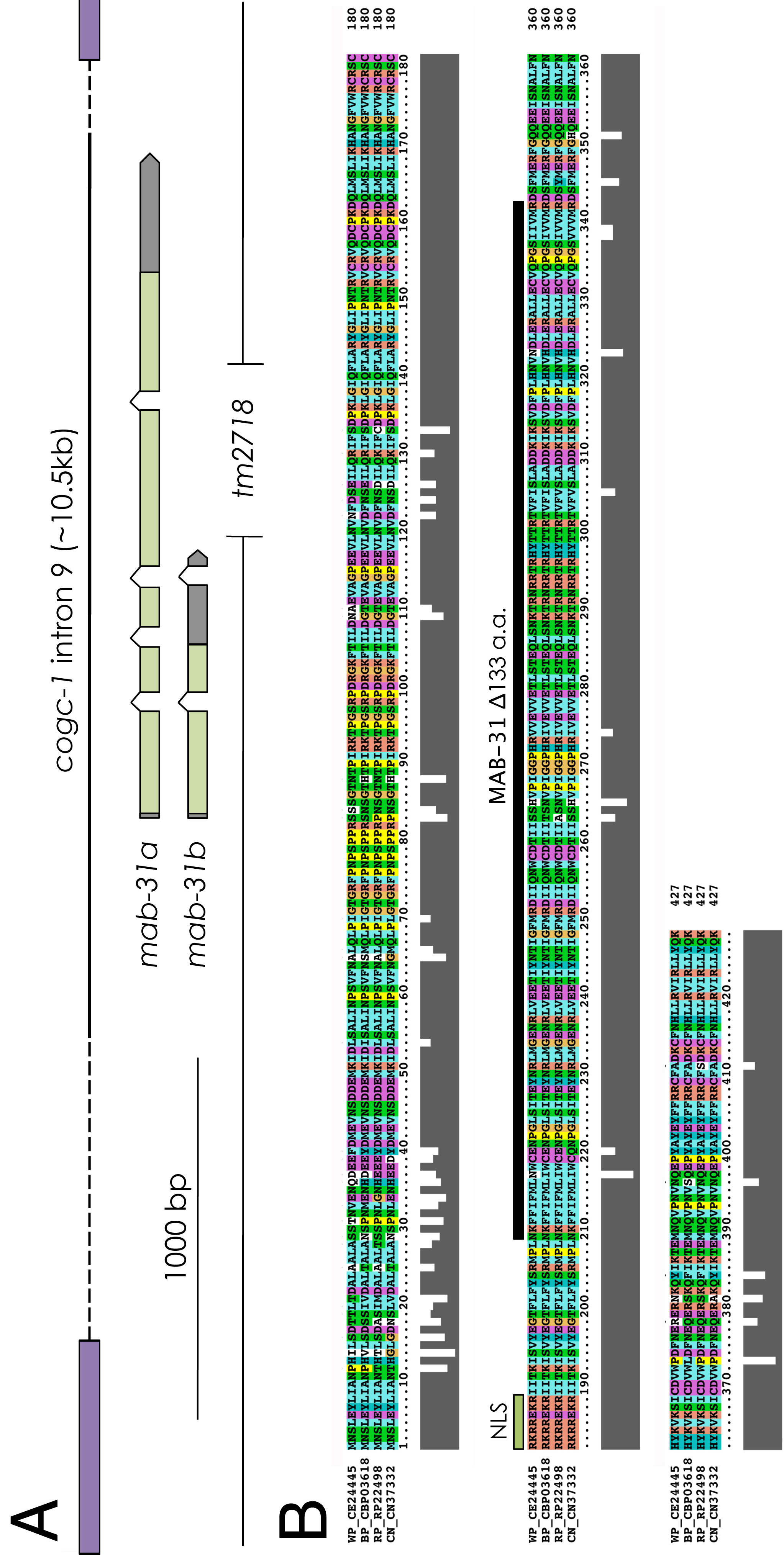

Supplement: Additional file 1 — Schematic representation of mab-31 gene structure and protein sequences. mab-31 gene products are embedded in ~10.5 kb intron 9 of cogc-1. Mutation tm2718 deleted coding sequence of mab-31a, but it does not affect the transcript level of mab-31b, a transcript irrelevant to the ray fusion phenotype. B) Sequence alignment of nematodes MAB-31 proteins (C. elegans: WP_CE24445, C. briggsae: BP_CBP03618, C. remanei: RP_RP22498, and C. brenneri: CN_CN37332) was shown by ClustalX. The conserved nuclear localization signal (NLS) (PredictNLS online) and deleted region of MAB-31 protein in tm2718 mutant were predicted and labeled. [file 1471-213X-10-82-S1.JPEG]
